# Impact of Preoperative Aspirin on Long-Term Outcomes in Diabetic Patients Following Coronary Artery Bypass Grafting: a Propensity Score Matched Study

**DOI:** 10.21470/1678-9741-2020-0313

**Published:** 2020

**Authors:** Sleiman Sebastian Aboul-Hassan, Tomasz Stankowski, Jakub Marczak, Maciej Peksa, Marcin Nawotka, Ryszard Stanislawski, Lukasz Moskal, Adam Lipowski, Michel Pompeu B. O. Sá, Romuald Cichon

**Affiliations:** 1 Department of Cardiac Surgery, Medinet Heart Center Ltd, Nowa Sol, Poland.; 2 Department of Cardiac Surgery, Sana-Heart Center Cottbus, Cottbus, Germany.; 3 Department of Cardiac Surgery, Trent Cardiac Centre, Nottingham University Hospital, Nottingham, United Kingdom.; 4 Department of Cardiac Surgery, Medinet Heart Center Ltd, Wroclaw, Poland.; 5 Department of Vascular Surgery, Nowa Sol Multidisciplinary Hospital, Nowa Sol, Poland.; 6 Division of Cardiovascular Surgery of Pronto Socorro Cardiologico de Pernambuco, PROCAPE, Universidade de Pernambuco, Recife, Pernambuco, Brazil.; 7 Department of Cardiac Surgery, Warsaw Medical University, Warsaw, Poland.

**Keywords:** Coronary Artery Bypass, Diabetes Mellitus, Aspirin, Incidence, Propensity Score

## Abstract

**Introduction:**

This study aimed to determine the effect of preoperative aspirin administration on early and long-term clinical outcomes in patients suffering from diabetes mellitus (DM) undergoing coronary artery bypass grafting (CABG).

**Methods:**

In this observational study, a total of 315 patients were included and grouped according to the time interval between their last aspirin dose and the time of surgery; patients who had been continued aspirin intake with last administered dose ≤ 24-hours before CABG (n=144) and those who had been given the last dose of aspirin between 24 to 48 hours before CABG (n=171).

**Results:**

Multivariable analysis showed that the continuation of preoperative aspirin intake ≤ 24 hours before CABG in patients with DM is associated with reduced incidence of 30-day major adverse cardiac and cerebral events (MACCE) (*P*=0.004) as well as reduced incidence of composite 30-day mortality/MACCE (*P*=0.012). During mean follow-up of 37±17.5 months, the unadjusted hazard ratio (HR) showed that aspirin ≤ 24 hours prior CABG in patients with DM significantly reduced the incidence of MACCE and composite of mortality/MACCE during follow-up (HR: 0.50; 95% confidence interval [CI]: 0.29-0.87; *P*=0.014 and HR: 0.61; 95% CI: 0.38-0.97; *P*=0.039, respectively). However, after propensity score (PS) matching, the PS-adjusted HR showed a non-significant trend towards the reduction of MACCE during follow-up (HR: 0.58; 95% CI: 0.31-1.06; *P*=0.081).

**Conclusion:**

Continuation of preoperative aspirin intake ≤ 24 hours before CABG in patients with DM is associated with reduced incidence of early MACCE, but without significant influence on long-term outcomes.

**Table:** 

Abbreviations, acronyms & symbols				
BMI	= Body mass index		NYHA	= New York Heart Association
CABG	= Coronary artery bypass grafting		ONCABG	= On-pump coronary artery bypass grafting
CAD	= Coronary artery disease		OPCABG	= Off-pump coronary artery bypass grafting
CAEs	= Cerebral adverse events		OR	= Odds ratio
CCB	= Calcium channel blockers		PCI	= Percutaneous coronary intervention
CLD	= Chronic lung disease		pRBC	= Packed red blood cells
CI	= Confidence interval		PS	= Propensity score
DM	= Diabetes mellitus		PVD	= Peripheral vascular disease
HR	= Hazard ratio		RAASI	= Renin angiotensin aldosterone inhibitors
LVEF	= Left ventricular ejection fraction		RCT	= Randomized controlled trial
MACCE	= Major adverse cardiac and cerebral events		SD	= Standard deviation
MI	= Myocardial infarction		SMD	= Standardized mean difference

## INTRODUCTION

Diabetes mellitus (DM) constitutes one of the major risk factors for developing cardiovascular diseases, most importantly, coronary artery disease (CAD)^[1]^. Patients burdened with DM are at increased risk of adverse cardiovascular events by two-fold to three-fold when compared to patients without DM^[2]^. Recent guidelines of the European Society of Cardiology/European Association for Cardio-Thoracic Surgery, or ESC/EACTS, on myocardial revascularization recommended coronary artery bypass grafting (CABG) as the method of choice in the treatment of patients with multivessel CAD and DM (Class I-a)^[3]^. Although CABG is the most effective revascularization therapy in these patients, a recent meta-analysis showed that pre-existing DM places patients at higher risk for inferior long-term postoperative outcomes^[4]^. This observation could be explained by several possible mechanisms including insulin resistance, release of fatty acids in addition to other metabolic phenomena that may in turn lead to a series of events such as endothelial dysfunction, oxidative stress, inflammation, and platelet hyper-reactivity, which affects the vascular wall and enhances the progress of atherosclerosis^[5]^. It is a well-established fact that aspirin due to its antiplatelet and anti-inflammatory function is beneficial for patients with CAD. The administration of aspirin within 48 hours following CABG is associated with a reduced incidence of major adverse cardiac and cerebral events (MACCE)^[6]^. Despite aspirin’s beneficial effect in patients following CABG, its continuation throughout the perioperative period or the time of its discontinuation prior to surgery remain ambiguous.

Current guidelines differ in their recommendations regarding the use of preoperative aspirin before CABG. While some recommend its continuation until the day of surgery^[7-8]^, others recommend its discontinuation at least a few days prior elective CABG to decrease the risk of bleeding^[9]^. On the other hand, a recent meta-analysis showed that the continuation of preoperative aspirin intake is associated with reduced early mortality as well as reduced incidence of perioperative myocardial infarction (MI) following cardiac surgery^[10]^. Studies have shown that platelet function gradually recovers within 72 hours reaching full recovery within 96 hours after aspirin cessation^[11-12]^. Therefore, withdrawal of aspirin a few days before CABG could potentially lead to a partial or complete recovery of platelet function which could in turn increase the risk of thrombo-occlusive events in patients with DM. Recent studies showed that the continuation of aspirin intake with its last dose within 24 hours before CABG is associated with improved postoperative outcomes in terms of mortality, MACCE, and acute kidney injury^[13-15]^. Despite abundant evidence generated by the recent studies or meta-analyses, none of them evaluated the impact of preoperative aspirin on postoperative outcomes following CABG in patients with DM. Therefore, we aimed to investigate the effect of preoperative aspirin administration on early and long-term clinical outcomes in patients suffering from DM and CAD undergoing CABG.

## METHODS

**Fig. 1:**
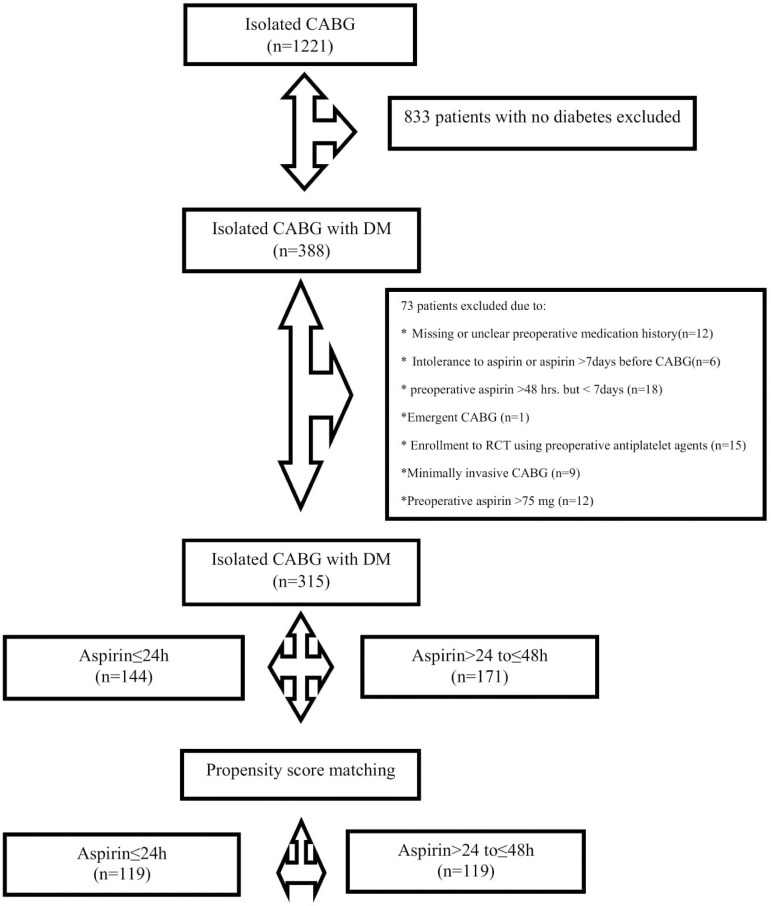
Patients’ flowchart diagram. CABG=coronary artery bypass grafting; DM=diabetes mellitus; RCT=randomized controlled trial

This observational, retrospective study was designed according to the Strengthening the Reporting of Observational Studies in Epidemiology, or STROBE, guidelines^[16]^. Between January 2014 and April 2018, 1221 patients underwent isolated CABG at the Department of Cardiac Surgery, Medinet Heart Center Ltd, Nowa Sol, Poland. Of these, 388 patients were diagnosed with type 2 DM. After analyzing and reviewing the data, 315 patients were included in the study and were divided into groups according to the time interval between their last aspirin dose and the time of surgery. Patients who had been continued aspirin with last administered dose ≤ 24 hours before CABG (n=144) and those who had been given the last dose of aspirin between 24 to 48 hours before CABG (n=171). Seventy-three patients were excluded due to: missing or unclear preoperative medication history (n=12), aspirin administration for more than 48 hours before CABG but less than seven days (n=18), documented intolerance to aspirin or did not take aspirin for more than seven days (n=6), emergency CABG (n=1), minimally invasive CABG (n=9), enrollment to ongoing randomized controlled trial (RCT) testing antiplatelet therapy following CABG (n=15), and administration of > 75 mg of aspirin before CABG (n=12). Figure 1 represents the patients’ flowchart diagram. The study was approved by the institutional review board at Medinet Heart Center and by the Bioethics Committee of Wroclaw’s Medical University, Poland. An individual consent of the patient for anonymous data analysis was waived by the Committee.

Patients’ demographics, clinical characteristics, medications, and postoperative outcomes were retrieved from the database of Medinet Heart Center, Nowa Sol, Poland. Preoperative aspirin dosing as well as the time of its last administration were both obtained from patients’ medication chart. The data covering preoperative dosage and time of the last aspirin dose taken by patients admitted to the hospital the day before surgery were obtained from the preoperative medication questionnaire. As for patients transferred from other departments, preoperative aspirin dosage and timing were obtained from patients’ discharge letter. Postoperatively, all patients received aspirin between six and 24 hours following CABG and that was continued daily thereafter. Follow-up data were retrieved from the national health care registry of the Ministry of Health of the Republic of Poland, that stores and analyzes all health-related data.

Study endpoints included early (30-day) and long-term MACCE and the composite of mortality and MACCE. Safety endpoints included postoperative chest tube drainage at 24 hours, postoperative transfusion rate of packed red blood cells (pRBC), need for re-exploration of the chest for bleeding, and duration of hospital stay. MACCE was defined as the occurrence of MI or cerebral adverse events (CAEs). MI was defined according to the third international definition of MI^[17]^. CAEs were defined as the development of a new permanent or transient (lasting up to 72 hours) neurologic deficit as confirmed by stroke team member assessment of the patient and computed tomography of the central nervous system, magnetic resonance imaging, or at autopsy examination.

Statistical analyses were performed using statistical software STATISTICA (TIBCO Software Inc. 2017, data analysis software system, version-13, Palo Alto, United States of America). Continuous variables were expressed as means±standard deviation (SD), while categorical variables as number and percentages. For continuous data, Student’s t-test or Mann- Whitney’s U-test was used for between groups comparisons, while categorical variables were compared with Pearson’s χ^2^ test.

To identify independent predictors of 30-day MACCE and composite of 30-day mortality and MACCE, we built a multivariable logistic regression model for the whole cohort by using all preoperative variables presented in Table 1. In addition to the above, operative and postoperative outcomes such as the type of surgery, number of grafts performed, duration of surgery, inotropic support, total arterial revascularization, and pRBC transfusion were included into the model. Multivariable logistic regression analysis was performed using stepwise backward regression. Only relevant covariates identified during univariate analysis with a *P*-value ≤ 0.1 were included in the final computation of a multivariable logistic model.

**Table 1:** Baseline characteristics of patients with diabetes undergoing coronary artery bypass grafting grouped according to time interval of aspirin discontinuation.

	Before matching			After matching			
	Aspirin		*P*-value	Aspirin		*P*-value	SMD
	≤ 24 h	> 24 to ≤ 48 h		≤ 24 h	> 24 to ≤ 48 h		
	(n=144)	(n=171)		(n=119)	(n=119)		
Age (years)	66.3±7.63	65.5±7.33	0.35	65.6±7.43	65.6±7.55	1	0.000
Female gender	37 (25.6)	62 (36.2)	0.045	36 (30.2)	34 (28.5)	0.77	0.044
PVD	34 (23.6)	39 (22.8)	0.86	27 (22.6)	29 (24.3)	0.75	
BMI (kg/m^2^)	30.1±4.81	30.3±4.37	0.70	29.9±4.89	30.2±4.21	0.61	
CLD	12 (8.3)	13 (7.6)	0.81	10 (8.4)	6 (5.0)	0.30	
LVEF (%)	48.7±9.50	49.6±10.29	0.42	49.3±9.19	49.1±10.2	0.87	0.020
History of CAEs	18 (12.5)	25 (14.6)	0.58	16 (13.4)	20 (16.8)	0.47	
Diabetes on insulin	79 (54.8)	91 (53.2)	0.77	61 (51.2)	67 (56.3)	0.43	
NYHA III-IV	18 (12.5)	14 (8.1)	0.20	16 (13.4)	10 (8.4)	0.21	
Recent MI (< 90 days)	48 (33.3)	62 (36.)	0.58	40 (33.6)	38 (31.9)	0.78	0.042
Urgent	86 (59.7)	92 (53.8)	0.29	72 (60.5)	64 (53.7)	0.29	
Previous PCI	27 (18.7)	45 (26.3)	0.11	23 (19.3)	33 (27.7)	0.12	
Hypertension	137 (95.1)	163 (95.3)	0.93	113 (94.9)	114 (95.7)	0.75	
Hyperlipidemia	95 (65.9)	110 (64.3)	0.76	76 (63.8)	75 (63.0)	0.89	
Active smoker	37 (25.6)	42 (24.5)	0.81	31 (26.0)	28 (23.5)	0.65	
N of diseased vessels	2.98±0.98	2.92±0.92	0.58	2.94±0.96	2.91±0.93	0.81	
Renal insufficiency*	79 (54.8)	101 (59.0)	0.45	67 (56.3)	67 (56.3)	1	0.000
**Preoperative medications ≤ 24 hours**							
B-blockers	101 (70.1)	92 (53.8)	0.003	76 (63.8)	75 (63.0)	0.89	0.019
CCB	11 (7.6)	13 (7.6)	0.99	8 (6.7)	9 (7.5)	0.80	
RAAS	40 (27.7)	43 (25.1)	0.59	32 (26.8)	36 (30.2)	0.56	
Statins	113 (78.4)	104 (60.8)	0.0009	88 (73.9)	85 (71.4)	0.66	0.07
Clopidogrel < 7days	14 (9.7)	26 (15.2)	0.14	12 (10.0)	19 (15.9)	0.18	

Data are expressed as mean±standard deviation or n (%), unless otherwise indicated.

* Renal insufficiency is defined as estimated glomerular filtration rate < 85 ml/min/1.73m^2^. BMI=body mass index; CAEs=cerebral adverse events; CCB=calcium channel blockers; CLD=chronic lung disease; LVEF=left ventricular ejection fraction; MI=myocardial infarction; NYHA=New York Heart Association; PCI=percutaneous coronary intervention; PVD=peripheral vascular disease; RAASI=renin angiotensin aldosterone inhibitors; SMD=standardized mean difference

To reduce the risk of selection bias inherent to retrospective, observational studies, a propensity score (PS) matching was used to match patients between the groups. PS were generated from a multivariable logistic regression model based on seven preoperative variables: age, gender, left ventricular ejection fraction, recent MI (< 90 days), renal insufficiency, and preoperative medication ≤ 24 hours (B-blockers and statins). Patients were then matched in 1:1 fashion using caliper matching method without replacement with a caliper width of 0.2 SD of the logit of PS^[18-19]^. The balance of the covariates was tested using standardized mean difference (SMD). Statistical guidelines suggest a meaningful covariate balance of the variables used to generate the PS between the two groups to be between -0.1 < SMD < 0.1^[18]^. Matched data were analyzed using procedures for matched analyses. For continuous data, Wilcoxon matchedpairs test was used, while McNemar’s test was used for binary outcomes.

Freedom from MACCE as well as freedom from composite mortality and MACCE at follow-up in the unadjusted and PSadjusted populations were estimated using the Kaplan-Meier method and were expressed as percentages. Log-rank test was used to compare the data. Hazard ratio (HR) in both populations with 95% confidence interval (CI) was derived from the Cox proportional hazards model. Statistical significance was defined as a *P*-value < 0.05.

## RESULTS

**Fig. 2:**
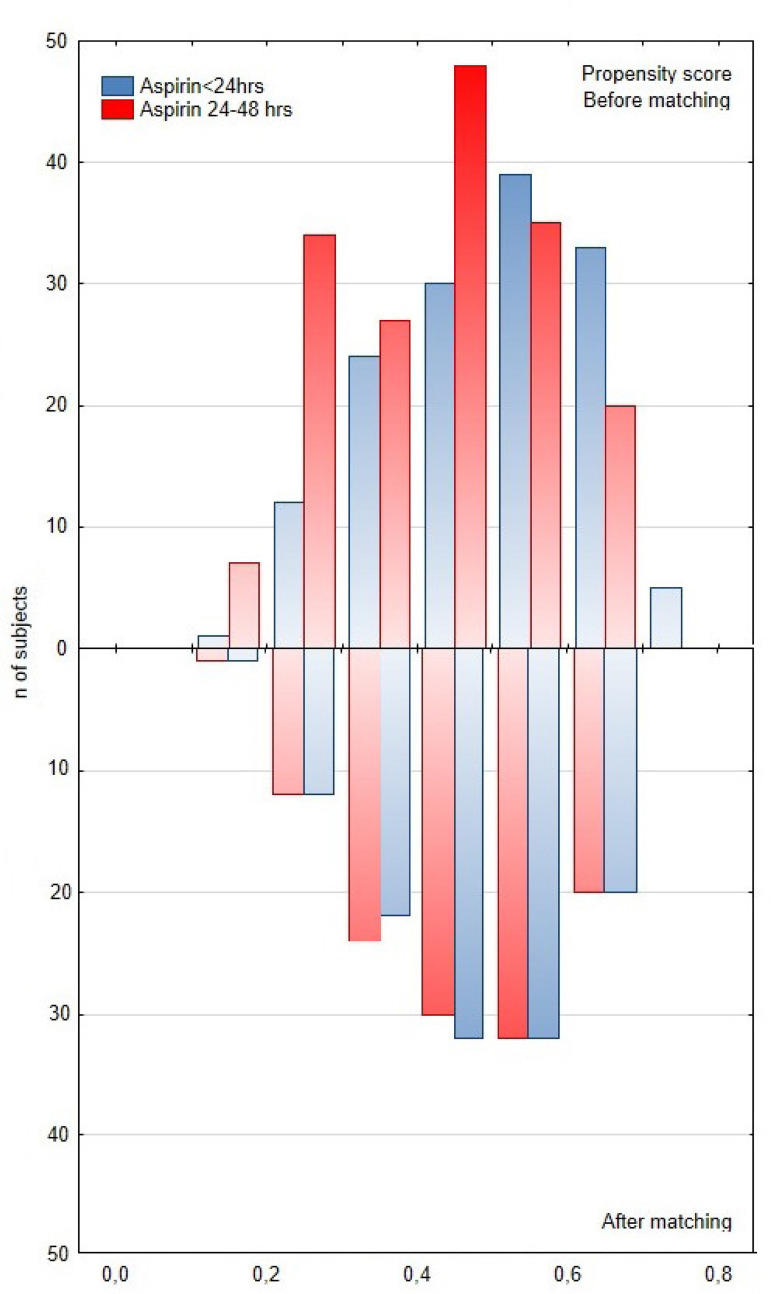
Mirrored histogram showing the propensity score distribution before and after matching in both groups.

Baseline and operative characteristics of the unadjusted and PS-adjusted populations are presented in Tables 1 and 2. In the unadjusted study groups, female patients were more likely to be found in the group where aspirin was discontinued between 24 and 48 hours before CABG, whereas patients in whom aspirin has been continued ≤ 24 hours before CABG were more likely to receive concurrent preoperative medication such as B-blockers and statins. There were no significant differences between both groups in terms of operative characteristics such as type of surgery, duration of surgery, number of grafts performed, and number of patients with total arterial revascularization (Table 2). After performing PS matching, the differences in baseline characteristics between both groups were eliminated (Table 1). PS matching selected 119 matched pairs for final comparison. The two matched groups were balanced (-0.1 < SMD < 0.1) (Table 1). Mirrored histogram showed adequate PS overlapping (Figure 2).

**Table 2:** Operative characteristics.

Type of surgery	Before matching			After matching		
	Aspirin ≤ 24 h	Aspirin > 24 to ≤ 48 h	*P*-value	Aspirin ≤ 24 h	Aspirin > 24 to ≤ 48 h	*P*-value
	(n=144)	(n=171)		(n=119)	(n=119)	
OPCABG	119 (82.6)	143 (83.6)	0.81	100 (84.0)	99 (83.1)	0.86
ONCABG	25 (17.4)	28 (16.4)	0.81	19 (16.0)	20 (16.9)	0.86
Duration (min)	168±53	167±51	0.86	169±53	165±48	0.48
Number of grafts	2.51±0.83	244±0.75	0.43	2.51±0.84	2.45±0.75	0.50
Total arterial revascularization	33 (22.9)	52 (30.4)	0.13	28 (23.5)	33 (27.7)	0.45

Data are expressed as mean±standard deviation or n (%), unless otherwise indicated. ONCABG=on-pump coronary artery bypass grafting; OPCABG=off-pump coronary artery bypass grafting

Multivariable analysis in the entire cohort showed that continuation of preoperative aspirin with the last dose administered within 24 hours before CABG in patients with DM is independently associated with reduced incidence of 30-day MACCE (odds ratio [OR]: 0.14; 95% CI: 0.04-0.54; *P*=0.004) as well as reduced incidence of composite 30-day mortality and MACCE (OR: 0.24; 95% CI: 0.08-0.73; *P*=0.012). Other significant predictors of 30-day MACCE were as follows: active smoking, use of inotropic support, and number of stenosed vessels. Age, inotropic support, and number of stenosed vessels were found to increase the probability of composite 30-day mortality and MACCE. After PS matching, patients with DM receiving aspirin within 24-hours prior CABG had a lower incidence of 30-day MACCE in comparison with patients who discontinued aspirin between 24 and 48 hours before CABG (3.3% *vs*. 10.0%, respectively, *P*=0.04) (Table 3). A non-significant trend towards reduced incidence of composite 30-day mortality and MACCE was observed in patients receiving aspirin ≤ 24 hours prior to CABG (4.2% *vs*. 10.9%, *P*=0.06) (Table 3). Before as well as after the PS matching, no significant differences were observed in terms of reoperation for bleeding, chest tube drainage, pRBC transfusions, and duration of hospital stay (Table 3).

**Table 3:** Early postoperative outcomes following coronary artery bypass grafting in diabetic patients.

	Before matching			After matching		
	Aspirin ≤ 24 h	Aspirin > 24 to ≤ 48 h	*P*-value*	Aspirin ≤ 24 h	Aspirin > 24 to ≤ 48 h	*P*-value ^+^
	(n=144)	(n=171)		(n=119)	(n=119)	
30-day mortality	1(0.69)	7(4.0)	0.09	1(0.84)	5(4.2)	0.22
30-day MACCE	4(2.7)	18(10.5)	0.01	4(3.3)	12(10.0)	0.04
Composite 30-day mortality/MACCE	5(3.4)	18(10.5)	0.02	5(4.2)	13(10.9)	0.06
Inotropic support	36(25)	44(25.7)	0.88	30(25.2)	30(25.2)	0.88
Reoperation for bleeding	7(4.8)	9(5.2)	0.87	7(5.8)	8(6.7)	1
Chest tube drainage at t=24 hours (ml)	646±314	610±291	0.29	635±316	605±309	0.31
pRBC transfusion	68(47.2)	73(42.6)	0.42	57(47.8)	45(37.8)	0.13
Duration of hospital stay (days)	7.1±2.48	7.6±3.9	0.18	7.1±2.6	7.8±4.3	0.14

Data are expressed as mean±standard deviation or n (%), unless otherwise indicated.

* Chi-square test or t-test.

+ McNemar test or Wilcoxon matched pairs test. MACCE=major adverse cardiac and cerebral events; pRBC=packed red blood cell

**Fig. 3:**
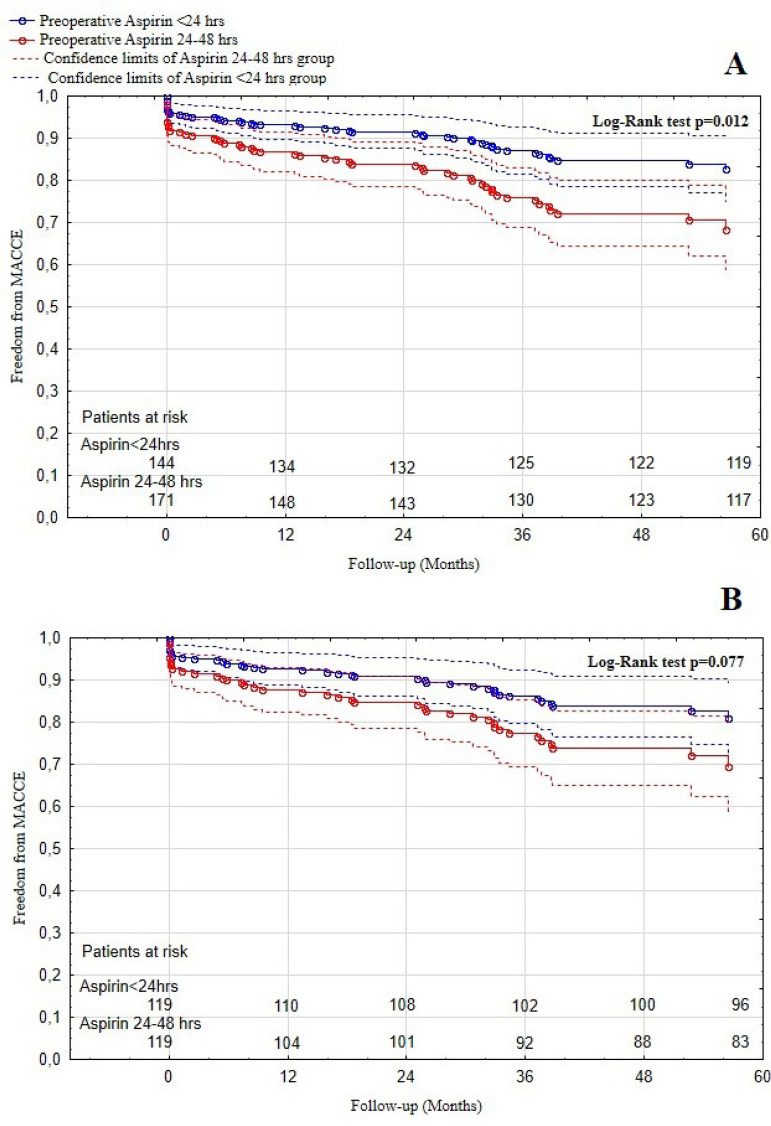
Kaplan-Meier freedom from major adverse cardiac and cerebral events (MACCE) curve. A) Before propensity score matching; B) after propensity score matching.

**Fig. 4:**
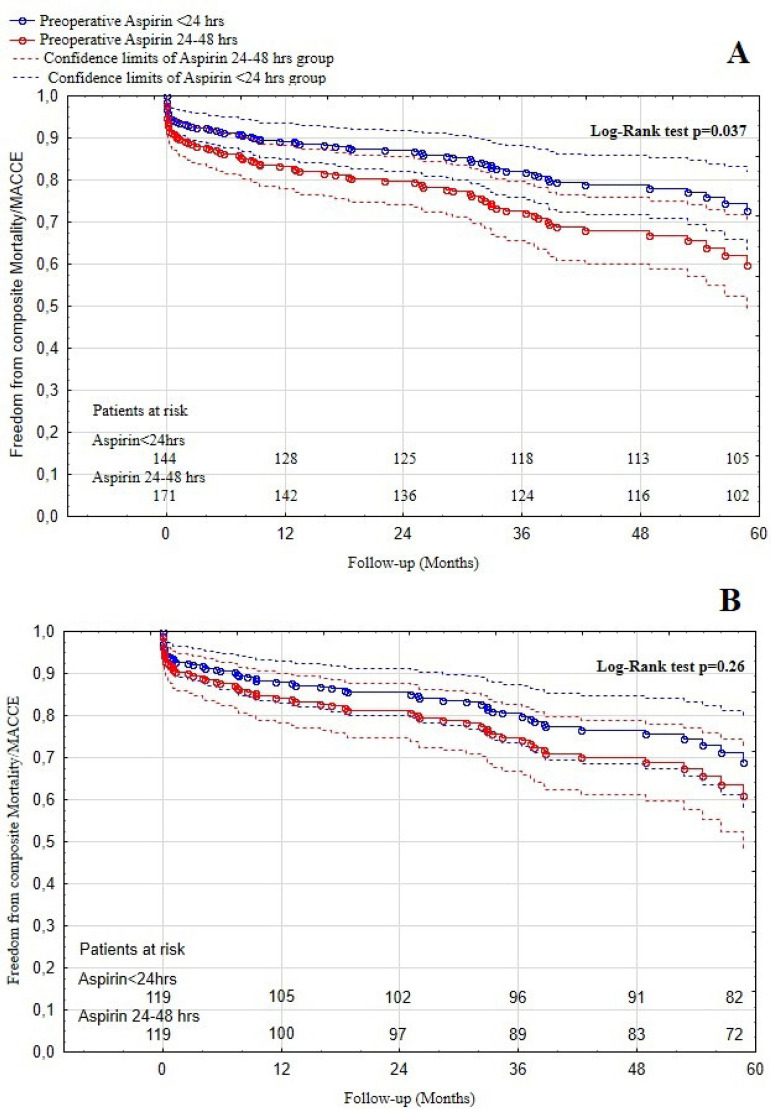
Kaplan-Meier freedom from composite mortality and major adverse cardiac and cerebral events (MACCE) curve. A) Before propensity score matching; B) after propensity score matching.

In the unmatched population, mean follow-up time was 37±17.5 months. Freedom from MACCE at 12, 36, and 60 months were 93.1%, 87.0%, and 82.5% in the aspirin ≤ 24-hour group and 86.7%, 75.8%, and 68.3% in the aspirin > 24-to-≤ 48-hour group (log-rank *P*=0.012, Figure 3A). Freedom form composite mortality and MACCE at 12, 36, and 60 months were 89.2%, 82.1%, and 72.7% in the aspirin ≤ 24-hour group and 83.1%, 72.7%, and 59.6% in the aspirin > 24-to-≤ 48-hour group (log rank *P*=0.037, Figure 4A). In the PS-matched cohort, the mean follow-up time was 38±17 months. Freedom from MACCE at 12, 36, and 60 months were 92.6%, 86.0%, and 80.8% in the aspirin ≤ 24-hour group and 87.7%, 77.3%, and 69.4% in the aspirin > 24-to-≤ 48-hour group (log rank *P*=0.077, Figure 3B). Whereas freedom from composite mortality and MACCE at 12, 36, and 60 months were 87.9%, 80.4%, and 68.9% in the aspirin ≤ 24-hour group and 84.2%, 74.7%, and 60.7% in the aspirin > 24-to-≤ 48-hour group (log rank *P*=0.26, Figure 4B). MACCE as well as composite of mortality and MACCE were assessed using Cox regression analysis. The unadjusted HR showed that aspirin ≤ 24 hours prior to CABG in patients with DM significantly reduced the incidence of MACCE and composite of mortality and MACCE during follow-up (HR: 0.50; 95% CI: 0.29-0.87; *P*=0.014 and HR: 0.61; 95% CI: 0.38-0.97; respectively; *P*=0.039). However, after PS matching, the PS-adjusted HR showed a nonsignificant trend towards reduced incidence of MACCE during follow-up (HR: 0.58; 95% CI: 0.31-1.06; *P*=0.081).

## DISCUSSION

DM is one of the main risk factors for CAD and is known to be associated with an increased rate of cardiovascular events when compared to patients with normoglycemia^[1-2]^. Endothelial dysfunction caused by DM triggers inflammation, which is associated with cardiovascular events in these patients^[20]^. Hyperglycemia creates a disproportion between nitric oxide and reactive oxygen species as well as reactive nitrogen species resulting in endothelial dysfunction^[21-22]^. Therefore, DM leads to vascular wall damage which in addition to platelet hyperreactivity enhances the progress of atherosclerosis, which in turn may contribute to prothrombotic conditions^[5]^. On the other hand, studies have shown increased thromboembolic events in patients undergoing CABG due to the use of extracorporeal circulation. On-pump CABG is known to induce inflammation and promotes endothelial injury^[23]^. While, in patients undergoing offpump CABG, a hypercoagulable state and platelet hyper-reactivity are observed^[24-26]^. Patients with DM undergoing CABG could be, therefore, at even higher risk for thromboembolic events as this was recently proven by a meta-analysis showing that DM constitutes a risk factor for worse long-term outcomes after CABG^[4]^. Aspirin with its antiplatelet and anti-inflammatory effects could act as a preventive measure for early thromboembolic events following CABG, especially in patients with DM, in whom aspirin was shown to have a protective effect not only for secondary prevention but also for primary prevention of first cardiovascular events^[27]^. The pharmacological mechanism of aspirin action is well described. Duration of the antiplatelet effect is determined by cyclooxygenase re-synthesis, which in case of matured platelets, its full replacement is needed to recover cyclooxygenase. However, studies have shown that the function of the platelets could gradually recover within 72 hours after aspirin withdrawal and fully normalizing its function within 96 hours^[11-12]^. Therefore, a partial recovery of the platelet function could be observed as soon as 24 to 48 hours after aspirin discontinuation.

A recent meta-analysis showed that the continuation of preoperative aspirin intake is associated with reduced early mortality as well as reduced incidence of perioperative MI following cardiac surgery^[10]^. On the other hand, recent studies showed that the continuation of aspirin intake with the last aspirin dose administered within 24 hours before CABG is associated with reduced early mortality as well as reduced incidence of MACCE^[13-14]^. Deja et al.^[28]^ in a well-designed RCT showed that the administration of preoperative aspirin prior CABG is associated with a decreased long-term hazard of MI or repeated revascularization (HR: 0.58; 95% CI: 0.33-0.99; P=0.046), whereas Xiao et al.^[29]^ observed a trend toward decreased midterm hazard of angina recurrence in patients in whom aspirin was continued till surgery. However, none of the available metaanalyses nor studies described the effect of preoperative aspirin in patients with DM undergoing CABG. Therefore, in this study, we aimed on determining the effect of preoperative aspirin use on early and long-term outcomes according to time intervals in patients with DM following CABG. We showed a clear benefit in patients with DM who took aspirin 24 hours or less prior CABG without increasing the risk of bleeding.

### Limitations

The present study has several limitations. Firstly, this was a single-center, retrospective study without randomization. To reduce the risk of bias, we used PS matching. However, despite its use, many factors may still affect outcomes. Secondly, we were unable to adjust for antifibrinolytic agents due to a systematic lack of its reporting. Thirdly, we had no influence on postoperative medication at follow-up and this factor could not be adjusted in long-term follow-up. Finally, we are aware that this study might be underpowered by the sample size. Therefore, large multi-center, randomized controlled studies are required in patients with DM undergoing CABG.

## CONCLUSION

In conclusion, in patients with DM undergoing CABG, continuation of preoperative aspirin intake till the day of surgery with the last dose administered within 24 hours or less prior CABG is associated with reduced incidence of early MACCE, but without significant influence on long-term outcomes.

**Table:** 

**Authors' roles & responsibilities**	
SSAH	Substantial contributions to the conception or design of the work; or the acquisition, analysis, or interpretation of data for the work; drafting the work or revising it critically for important intellectual content; final approval of the version to be published
TS	Drafting the work or revising it critically for important intellectual content; final approval of the version to be published
JM	Interpretation of data for the work; drafting the work or revising it critically for important intellectual content; final approval of the version to be published
MP	Drafting the work or revising it critically for important intellectual content; final approval of the version to be published
MN	Drafting the work or revising it critically for important intellectual content; final approval of the version to be published
RS	Drafting the work or revising it critically for important intellectual content; final approval of the version to be published
LM	Drafting the work or revising it critically for important intellectual content; final approval of the version to be published
AL	Drafting the work or revising it critically for important intellectual content; final approval of the version to be published
MPBOS	Drafting the work or revising it critically for important intellectual content; final approval of the version to be published
RC	Drafting the work or revising it critically for important intellectual content; final approval of the version to be published
